# Organ communication in ageing

**DOI:** 10.1098/rstb.2025.0333

**Published:** 2026-11-12

**Authors:** Giulia Allavena, Hervé Técher, Miguel Godinho Ferreira

**Affiliations:** Institute for Research on Cancer and Aging Nice (IRCAN), CNRS UMR7284, INSERM U1081, Université Côte d'Azur, Nice, France

**Keywords:** ageing, organ-to-organ communication, senescence, cGAS–STING, telomere attrition

## Abstract

Ageing is a systemic process in which local damage in one organ propagates to others and coordinates organism-wide decline. The hallmarks of ageing are established, but the causal relations between them, and the routes by which local damage becomes organismal decline, remain undefined. We propose an initiator–communicator–effector framework in which the three terms denote causal roles assigned by intervention rather than fixed classes of lesion. *Initiators* are organ-intrinsic lesions whose organ-restricted correction restores function in distant, untreated organs; telomere attrition in the zebrafish gut is the paradigm. *Communicators* are the organs, routes and mediators whose interruption uncouples distant phenotypes from the initiating lesion without correcting it, including sterile inflammation, cGAS–STING signalling, endocrine and metabolic factors, immune-cell trafficking and neuroimmune outputs. *Effectors* are states induced in recipient organs lacking a sufficient local lesion, notably stem-cell exhaustion, bioenergetic decline, epigenetic reprogramming, fibrosis and endothelial dysfunction. Because roles are defined by perturbation rather than molecular identity, the same process may initiate on one axis and act as an effector on another; the architecture is a directed graph containing feedback loops, not a linear chain. Ageing may therefore be slowed by interrupting chronically engaged organ-to-organ communication as well as by repairing initiating damage.

This article is part of the theme issue ‘Developmental circuits in ageing’.

## 1. Introduction—ageing as a network of communicating organs

Ageing is classically defined as a progressive, time-dependent decline in physiological function that increases vulnerability to disease and death [1]. Over the last decade, this decline has been deconstructed into a set of ‘hallmarks’ of ageing, including genomic instability, telomere attrition, loss of proteostasis, deregulated nutrient sensing, mitochondrial dysfunction, stem-cell exhaustion and altered intercellular communication [2]. This framework has been extremely influential in organizing the field and in guiding the search for molecular targets. What it does not yet specify is the causal architecture linking the hallmarks to one another, nor the routes by which damage confined to one organ becomes a decline of the organism; supplying that architecture, and a set of criteria by which it can be tested, is the aim of the framework proposed here. Most hallmarks have been studied primarily within single tissues or cell types and are often conceptualized as cell-autonomous processes [3]. In contrast, clinical observations and systems-level analyses increasingly indicate that ageing is fundamentally systemic: local perturbations in one organ can propagate to others and coordinate organism-wide functional decline [4,5].

A parallel body of work in invertebrate and vertebrate models has developed the idea of an ‘inter-organ communication network’, in which endocrine, metabolic, immune and neural signals link distant tissues into a dynamic physiological ageing unit [6]. In flies and worms, manipulation of specific tissues, such as skeletal muscle, intestine, neurons or adipose tissue, can non-cell-autonomously extend or shorten lifespan, demonstrating that ageing can be driven by a limited number of ‘control nodes’ rather than being purely the sum of independent cellular clocks [7]. In mammals, interventions targeting adipose tissue, liver, hypothalamus or haematopoietic stem cells similarly remodel systemic metabolism and inflammatory tone with consequences for whole-organism ageing [8]. Together, these findings suggest that the canonical hallmarks should be revisited considering organ-to-organ communication: damage that is initially local can be converted into systemic programmes that influence ageing across multiple tissues.

To formalize this view, it is useful to distinguish three conceptual levels: initiators, communicators and effectors (figure 1). First, *initiators* are organ-intrinsic processes that progressively cross a functional threshold with age and create local damage. We use the term as a causal role rather than as a class of lesion: a process occupies the initiator role on a given axis when its correction, restricted to one organ, improves function in distant organs that receive no direct intervention, and when its induction, similarly restricted, is sufficient to degrade them (box 1). Nor do all initiators accumulate monotonically. Barrier permeability and dysbiosis are threshold phenomena and are in principle reversible, whereas telomere attrition in the absence of telomerase is not, a distinction that determines which initiators are tractable targets. Candidate initiators include telomere attrition and other forms of genomic instability, persistent DNA damage responses, mitochondrial dysfunction and altered organelle quality control, as well as barrier failure and microbiota alterations at externally exposed environments of the gut, skin and lung [11,12]. These initiators arise in specific anatomical contexts and may differ in timing and intensity between organs, introducing heterogeneity in when and where ageing begins.

**Figure 1.**
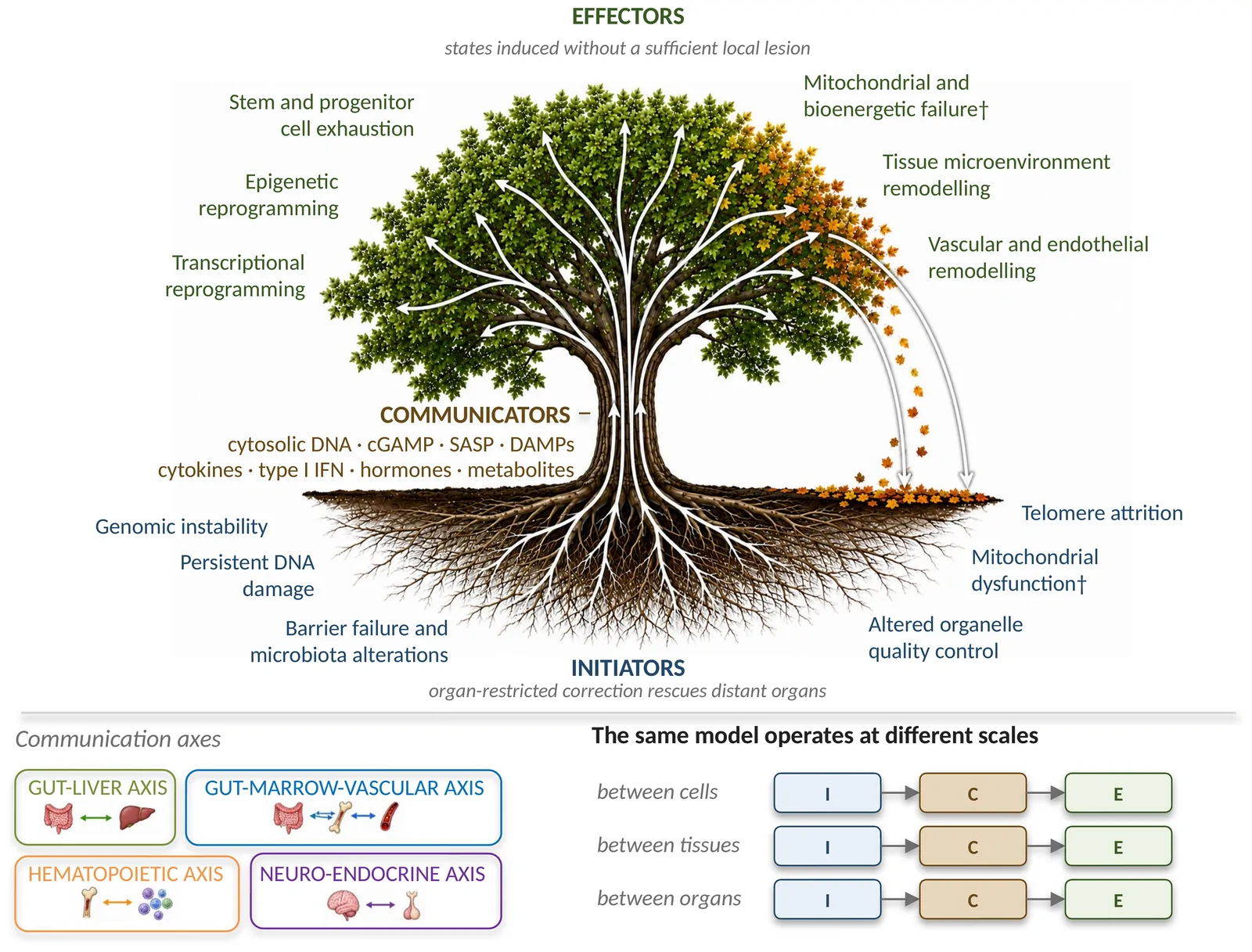
Conceptual ‘initiator → communicator → effector’ diagram, with the four communication themes mapped onto the communicator layer. Roles are assigned by the interventional criteria of box 1 and are axis-relative. The tree is read from the roots upwards: initiators arise locally, are relayed through the communicator trunk and are expressed as effector states in the canopy. The white arrows mark this dominant direction of causal flow, with tiers separated at the soil surface and at the base of the crown. The descending trail of senescing leaves on the right marks the return path from the effector to the initiator layer. Together, the two make the architecture a directed cyclic graph in which edges differ in weight and latency, not a linear hierarchy. The four communication axes are shown below the tree, and the inset shows the same motif operating between cells, between tissues and between organs. Mitochondrial dysfunction is marked with a dagger symbol where it appears in both the initiator and the effector tier: this is the worked example of role-relativity set out in box 1, not an inconsistency.

**Box 1. Operational criteria for assigning causal roles**

Roles are assigned by intervention, not by molecular identity, and always with respect to a defined donor→recipient axis.

*Initiator.* (i) The process arises cell-autonomously within a defined anatomical compartment; (ii) its correction restricted to that compartment improves function in distant compartments receiving no intervention; and (iii) its induction restricted to that compartment is sufficient to degrade function in distant compartments.

*Communicator.* Interruption of the route uncouples the recipient phenotype from the donor lesion without correcting the donor lesion.

*Effector.* The state is induced in the recipient compartment in the demonstrable absence, or insufficiency, of a local initiating lesion.

Three consequences follow. Roles are axis-relative and reversible. The criteria are falsifiable at edge level, since each is a perturbation rather than a matter of definition. Most candidate processes have not yet been tested against them and are therefore unassigned rather than assigned by assumption. This is the organ-scale form of the driver-versus-passenger problem [9,10].

Second, *communicators* are the organs and tissues through which local damage is spread to the rest of the organism, together with the routes and mediators they generate. The organ is the anatomical unit of the framework; the SASP (the senescence-associated secretory phenotype), cytosolic nucleic acid sensing, endocrine outputs and trafficking immune cells discussed below are the means by which an organ broadcasts and are communicators in that sense. Operationally, a route occupies the communicator role when its interruption uncouples the distant phenotype from the initiating lesion without correcting the lesion itself (box 1). When an epithelial barrier becomes leaky, a liver fibrotic, a bone marrow myeloid-biased or a heart hypertrophic, organ-scale structural changes affect the bidirectional exchange with the circulatory system and thereby amplify and disseminate their local failure [13,14]. In this sense, gut, liver, adipose tissue, kidney, bone marrow or brain act as anatomical ‘broadcast stations’: their structural and functional deterioration increases the flux of mediators and alters haemodynamics, innervation and lymphatic drainage, turning chronic organ damage into a sustained outgoing signal [15]. At the molecular level, they are composed of soluble inflammatory mediators, such as SASP produced by senescent cells, cytosolic nucleic acids and other DAMPs (damage-associated molecular patterns), that activate innate immune pathways, such as cGAS–STING, microbiota-derived molecules and metabolites, that transit via the portal and systemic circulation, endocrine factors (hormones, growth factors, lipokines, myokines and hepatokines), circulating metabolites that report on nutritional and energetic status, and the trafficking of immune cells whose transcriptional and epigenetic states have been modified by aged tissues [16,17]. Neuroimmune pathways, in which autonomous and sensory neurons detect peripheral stress and centrally adjust sympathetic, parasympathetic and neuroendocrine outputs, provide an additional fast layer of communication [18]. Because these communication routes are anatomically localized (often passing through discrete organs such as the intestine, liver, bone marrow or hypothalamus), they represent points at which the transmission of ageing signals can be attenuated or blocked. This is not a speculative claim: gut-restricted telomerase re-expression and loss of STING each protect distant organs in telomerase-deficient zebrafish despite persistence of the initiating lesion (§§3b and 5c), decoupling the fate of secondary organs from that of the initiating tissue.

Third, *effectors* are the relatively limited set of cellular and tissue-level states, the term being used in its physiological sense of a downstream functional output rather than of a molecule, that are repeatedly altered across organs in response to the communication layer. Operationally, a process occupies the effector role when it is induced in a recipient organ in the demonstrable absence, or insufficiency, of a local initiating lesion (box 1). Key effectors include the following: (i) stem and progenitor cell exhaustion and disruption of their niches; (ii) mitochondrial and bioenergetic failure with increased reactive oxygen species (ROS) and reduced flexibility; (iii) stable epigenetic and transcriptional reprogramming that locks cells into inflammatory or senescent states; (iv) structural remodelling of tissue microenvironments and vasculature, such as fibrosis, matrix stiffening and endothelial dysfunction [19,20]. The central hypothesis of this review is that diverse initiators, acting through identifiable organ-to-organ communication, converge on this effector layer to produce systemic ageing, and that intervening at connection points can decouple the fate of secondary organs from that of the initiating tissue.

Three properties of this scheme should be stated at the outset. First, the three terms designate roles, not kinds. Because assignment is made by experimental perturbation rather than by molecular identity, the same process may act as an initiator on one axis and as an effector on another. Mitochondrial dysfunction is the clearest case, initiating in muscle and adipose tissue while behaving as a downstream effector in organs that receive inflammatory and interferon input. Epigenetic and transcriptional reprogramming and microbiota alterations are axis dependent in the same way, and we leave them unassigned rather than fix them to one tier. Second, the resulting architecture is a directed graph that contains cycles rather than a linear chain: effector states feed back onto the initiator layer (figure 1 and §6b). Third, the motif is scale-invariant, and analogous initiator–communicator–effector relations operate between cells within a tissue and between tissues within an organ. We centre the description on organs because that is the scale at which perturbation can be anatomically restricted *in vivo*, as in gut-specific telomerase re-expression, and at which organ-resolved epigenetic and proteomic clocks now make the question decidable in humans [21,22].

## 2. Senescent and damaged cells as initiators of cytoplasmic DNA-driven inflammatory communication

### (a) Telomere attrition, DNA damage and senescence in ageing as initiators

Telomeres are nucleoprotein structures capping the repetitive sequences located at the extremities of linear chromosomes [23–25]. In early development and embryonic cells, telomeres are elongated by telomerase (encoded by Tert). Later in adult life, and in most somatic cells, telomerase expression is lost and telomeres shorten with age as a result of cell divisions. Telomeres become critically short with ageing across different species, including humans [26,27], zebrafish [24,28] and other vertebrates [29].

When telomeres are eroded, cells stop dividing, reaching a proliferative exhaustion, the so-called replicative senescence [30]. Indeed, short telomeres are structurally similar to DNA double-strand (ds) breaks (DSBs), activating the ATM–CHK2–p53 axis of the DNA damage response (DDR) that promotes senescence [31]. The main characteristic of senescence is a stable or permanent exit from the cell cycle. Pre-senescent and senescent cells express p21 (CDKN1A) and/or p16 (CDKN2A), which are cell-cycle inhibitors. Senescent cells accumulate with time in vertebrates, including humans [32]. Still, their exact contribution to ageing and the molecular mechanisms involved in establishing senescence remain a central question in the field.

Are short telomeres possible ageing initiators? Successive in-crossing of Terc^−/−^ and Tert^−/−^ mice leads to short telomeres and is associated with premature-ageing phenotypes [33–36]. Indicating that short telomeres can initiate ageing in mammals. In zebrafish, the first generation of *tert* mutants have short telomeres, premature ageing and reduced lifespan, as early as 12 months, compared to an average of 3–4 years of life in wild-type (WT) zebrafish [28,37,38]. Notably, telomere shortening in *tert* mutant zebrafish occurs concomitantly to the accumulation of senescent cells and decline in proliferation [28,37,39,40]. Similarly, shortening of telomeres correlates with the accumulation of senescent cells and biological age in humans [41]. Ageing is likely to be initiated by different types of DNA lesions and chromosomal defects, affecting not only telomeres but also other genomic regions. For instance, several types of stresses and DNA-damaging conditions, including irradiation or oncogenic activation, all lead to senescence [42]. Therefore, chromosomal instability is a major trigger of senescence. Cells from old individuals, when compared to cells from young donors, harbour more chromosomal instability, mainly owing to mitotic segregation defects [43,44]. Notably, ‘old cells’, experiencing DNA damage and DNA replication defects, accumulate micronuclei [45], a piece or whole chromosome that is physically separated from the primary nucleus after unsuccessful mitosis [46,47].

### (b) The senescence-associated secretory phenotype and paracrine senescence as communicators

The stable proliferation arrest of senescent cells is accompanied by the secretion of pro-inflammatory cytokines, chemokines and growth factors, known as the SASP [48]. While SASP composition varies across tissues and contexts, its effect is to reshape local microenvironments and to communicate the presence of damage. Overexpression of p21 and p16, although arresting cell proliferation, is not sufficient to promote the SASP [49]. The SASP is not produced only by proliferation arrest but is rather a communication of damage.

The secretion of inflammatory signals by senescent cells has been proposed to maintain and spread senescence, in an autocrine and paracrine manner, respectively. Indeed, the incubation of normal cells with conditioned medium of senescent cells, or the co-culture of normal and senescent cells, all resulted in turning normal proliferating cells into senescent cells [50,51]. Thus, the secretome of senescent cells (e.g. TGF-b, IL6 and IL1) can communicate the senescence status to other cells. In mice, IL11, a constituent of SASP and of IL1-signalling, appears to be a critical driver of ageing [52]. It was shown that challenging cells with IFN-β is sufficient to induce senescence [51,53]. Importantly, paracrine senescence induced by conditioned medium or exposing cells to prolonged treatment with IFN-β were both sufficient to induce DNA damage. An important contributor of paracrine senescence is therefore the ability by which sterile inflammation or SASP spreads a stressing and damaging signal, altering genome stability in the microenvironment or beyond. Paracrine senescence has been consistently observed *in vivo* in mouse models and human samples [51]. Sterile inflammation from senescent cells can therefore spread in a paracrine manner and be a communicator. This hypothesis is supported by experiments showing that connecting old to young mice by the blood system, in a ‘parabiosis’ system, leads to the accelerated ageing of the young mice [54,55]. Parabiosis establishes that blood-borne exchange can transmit age-associated effects but does not by itself separate soluble mediators from exchanged circulating cells. These types of experiments indicate that SASP/sterile inflammation from old tissues can communicate and spread ageing, contributing to DNA damage accumulation and senescence during lifetime.

### (c) Cytoplasmic DNA as a central initiating signal

SASP is mainly mediated by the cGAS–STING pathway [50,56–59]. Cytoplasmic DNA or RNA:DNA hybrids are sensed by the cyclic GMP-AMP synthase (cGAS), which activates the stimulator of interferon genes (STING) [60]. STING activation triggers TBK1 and IRF3 phosphorylation, leading to type I interferon (IFN-I) production, induction of interferon-stimulated genes (ISGs) and chronic NF-κB activation. These programmes not only act locally but also reshape distant tissues through circulating inflammatory signals. STING–IFN-I signalling and SASP therefore provide a mechanistic bridge between intracellular damage and systemic communication [50].

The nucleic acids that activate this pathway during ageing likely arise from several recurrent forms of DNA damage, including micronuclei or cytoplasmic chromatin fragments, mitochondrial nucleic acid leakage and derepression of retrotransposable elements [50,56–59,61,62]. Rather than treating these substrates as competing explanations, it is useful to view them as different consequences of genomic instability, organelle dysfunction and epigenetic drift that converge on the same inflammatory output. Whatever their origin, cytoplasmic nucleic acids intensify sterile inflammation and can amplify senescence in neighbouring and distant cells [50,51,56].

Recent mammalian genetics supports this causal ordering. Failure of DNA–protein cross-link repair in a Sprtn knock-in mouse model of Ruijs–Aalfs progeria produces chromosome segregation errors, micronuclei and cytosolic nucleic acid release, and the resulting chronic cGAS–STING signalling is what drives embryonic lethality and progeroid phenotypes: genetic or pharmacological inhibition of cGAS–STING rescues both [63]. Conversely, STING inhibition in aged mice reduces neuroinflammation and improves cognition [64]. In terms of box 1, the cross-link lesion is the initiator and cGAS–STING the communicator: inhibiting the pathway uncouples the progeroid phenotype from the lesion without repairing a single cross-link. Whether nucleic acid sensing holds this role on telomere dysfunction in mammals is now contested. In telomerase-deficient mice, constitutive STING deletion fails to rescue degenerative pathology, infertility or lifespan in either Terc- or Tert-null backgrounds, and the authors conclude that cGAS–STING is not the principal driver of telomere-dependent phenotypes in that model [65]. In the terms used here, that is a statement about drivers rather than about communicators, and the two are distinct [9,10]: the communicator criterion of box 1 is defined on a donor→recipient axis, whereas a constitutive whole-animal deletion removes the sensor everywhere at once, including in tissues where it is protective, so pathological and surveillance contributions cannot be separated. A communicator also amplifies rather than originates, and amplification requires a localised primary lesion to supply continuous substrate. Mice and fish (and humans) differ in telomere length, in barrier biology and in which organ crosses threshold first, and the mouse experiment does not identify a dominant initiator organ to interrupt. Organ-restricted rather than whole-animal interruption is therefore the discriminating test, and it has not yet been performed in mammals. That role is also conditional. Where a persistent lesion supplies continuous cytosolic DNA, interrupting the pathway protects distant organs. In naive tissue, the same pathway guards the genome. Nuclear cGAS maintains heterochromatin and restrains retroelement activity, and cGAS-deficient mice show LINE1 derepression, DNA damage, inflammation and a premature-ageing phenotype [66]. Communicator status therefore belongs to an axis rather than to a molecule, and the contrast between lesion-loaded and naive backgrounds is what tests it. Zebrafish provide a compelling demonstration of how the cGAS–STING pathway governs systemic ageing in telomerase-deficient fish [39,67]. Telomere erosion increases cytosolic DNA, activates STING-interferon signalling and drives premature multi-organ ageing. Genetic deletion of STING rescues proliferation, fertility and lifespan despite persistent telomere attrition, indicating that the key driver of systemic decline is not genomic instability *per se* but its translation into chronic inflammatory signalling [39].

## 3. Gut–microbiome–liver–haematopoietic–vascular as a central communication axis

### (a) Barrier failure, dysbiosis and outgoing information

The intestine is anatomically positioned to act as a primary communication organ between the external environment and internal tissues. A single epithelial layer, supported by stem cells and a complex immune and nervous system, holds an extensive vascular and lymphatic network that drains first to the liver and then to the systemic circulation and bone marrow [68]. With age, this structure undergoes distinctive deterioration: epithelial turnover becomes faulty, villi architecture is altered, tight junctions are weakened, mucus and antimicrobial defences deteriorate, and local immune cells adopt chronically activated states [69]. These changes collectively constitute an organ barrier dysfunction.

Ageing is accompanied by alterations in the intestinal microbiota, including reduced diversity, with loss of health-associated taxa, expansion of opportunists and low-grade mucosal inflammation [70,71]. Gut barrier failure alters the ecological niche, favouring microbial communities that tolerate or reinforce inflammation, while dysbiosis feeds back on epithelial and immune cells to further erode barrier integrity [72]. Experimental transfer of aged microbiota into young recipients is sufficient to induce intestinal stress and barrier weakening, whereas young microbiota can partially restore epithelial homeostasis in old hosts, indicating that the communication can be modified independently of the host age [73].

The liver is the downstream recipient of intestinal venous drainage through the portal vein and therefore acts as the first anatomical hub for systemic communication. Hepatic sinusoidal endothelial cells, resident macrophages and stellate cells continuously respond to the gut [74]. In young organisms, this results in a largely tolerance-prone response that maintains metabolic and immune homeostasis. With age, chronic exposure to gut leakiness shifts the liver towards sustained inflammation, steatosis and fibrosis, thereby filtering, amplifying and re-encoding intestinal signals into systemic immune and metabolic outputs [75].

Bone marrow and its niches are exquisitely sensitive to chronic inflammatory and metabolic inputs. In the context of gut communication, the marrow receives blood conditioned by the intestine and transformed by the liver. These combined signals influence haematopoietic stem-cell proliferation, lineage bias and self-renewal, as well as stromal and endothelial niche behaviour [76]. Increased haematopoietic turnover results in telomere shortening and immunosenescence [77], yielding a compartment biased towards chronically activated, pro-inflammatory monocytes, macrophages and T-cells that circulate widely and communicate ageing information to distant tissues [78].

### (b) Proof of principle: gut-restricted telomerase rescue in zebrafish

The strongest test of the framework is an intervention confined to a single organ that changes the ageing of others; vertebrate models allow that test to be performed directly. The telomerase-deficient zebrafish are a particularly informative example [24]. In this model, telomeres shorten prematurely, and DNA damage accumulates across multiple tissues [28]. However, the earliest defects occur in the zebrafish gut [28,67]. Lack of telomerase results in telomere shortening, telomere damage-induced foci (TIFs), loss of cell proliferation, increased cell senescence, gut barrier breakdown, microbiota dysbiosis and mucosal inflammation [28,39,67,79]. Other proliferative organs, such as testis and kidney marrow, present shorter telomeres and reduction in cell proliferation, while TIFs are not observed and only later display overt dysfunction [28,67].

When telomerase is re-expressed specifically in the gut of telomerase-deficient zebrafish, intestine telomeres are elongated, epithelial proliferation and barrier integrity recover, and the microbiota shifts towards a more youthful composition [67]. Strikingly, this gut-restricted intervention is sufficient to improve fertility and delay gonadal atrophy, preserve haematopoietic architecture and function, reduce systemic inflammation and extend the median lifespan by 40%. However, telomeres in distant organs remain short, though markers of DNA damage and senescence decline, cell proliferation resumes, and tissue structure is maintained [67]. Thus, each organ does not age autonomously according to its own telomere length. Instead, telomerase-deficient gut functions as a primary communicator organ whose failure drives systemic ageing (figure 2). Correcting this defect, without modifying telomere length elsewhere, reduces the pathological signals in bone marrow and gonads [67]. In practical terms, and in this dominant-lesion context, blocking or normalizing a single initiator organ can maintain youthful states in secondary organs despite persistent defects.

**Figure 2.**
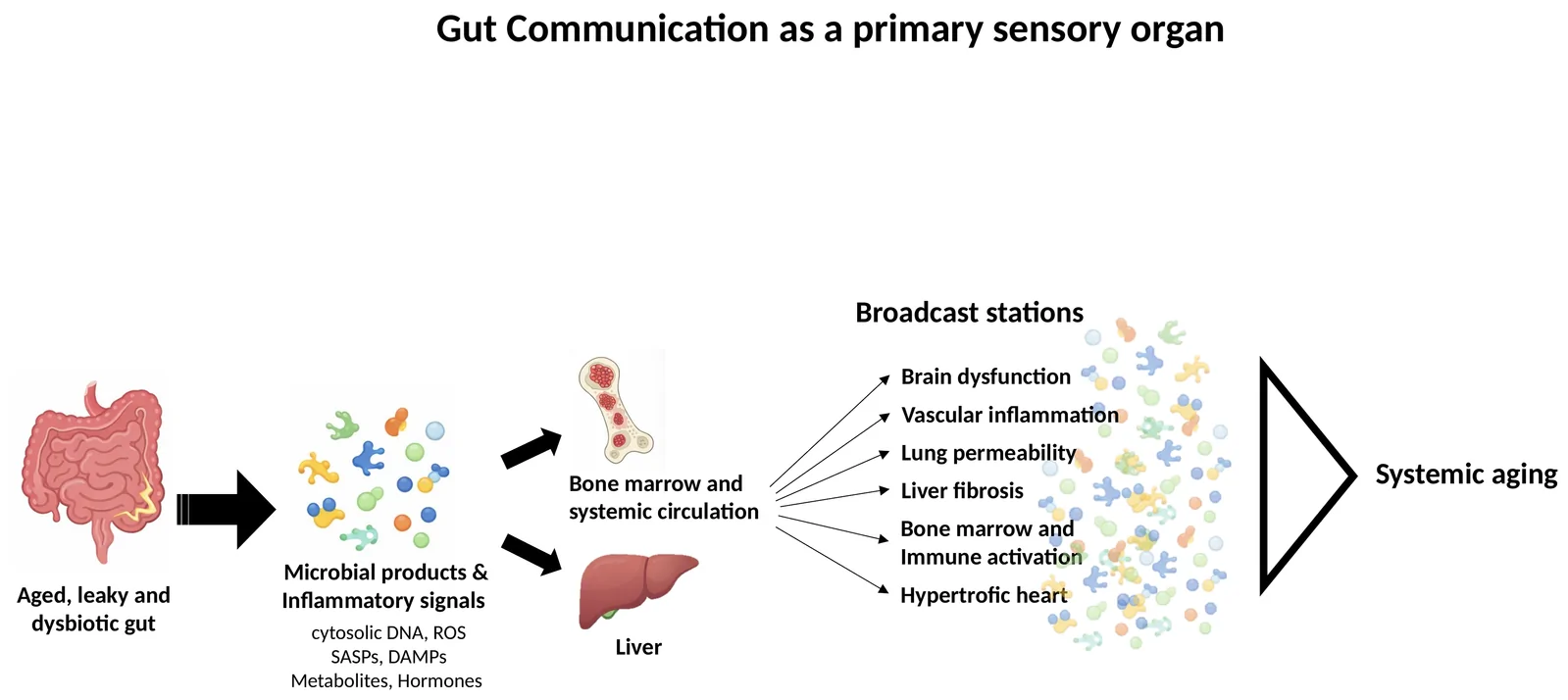
Schematic of gut–microbiome–liver–haematopoietic axis: intestinal damage/dysbiosis → microbial and metabolite signals → liver and bone marrow → systemic immune and metabolic changes.

## 4. Ageing immune system, metabolic/endocrine and neuroimmune communication

### (a) Intestinal immune cells as mobile communicators

The haematopoietic system occupies a distinctive position in the initiator–communicator–effector model because it is both shaped by upstream organ dysfunction and capable of distributing ageing information throughout the body [80]. With age, haematopoietic stem cells lose regenerative fitness, become biased towards myeloid output, show reduced lymphoid competence and are increasingly constrained by a remodelled bone marrow niche [81]. These changes, together with chronic inflammatory exposure and clonal haematopoiesis, favour immune cells with heightened basal inflammatory tone and impaired adaptive function [82,83].

A central implication is that immune cells function as mobile communicators of tissue age. Circulating and tissue-resident immune cells acquire transcriptional, epigenetic and metabolic states from the local environments they pass through and are then redistributed through blood and lymph, carrying those states with them [84]. This provides a mechanistic route by which initiator lesions in one organ can be amplified systemically even when the original insult is spatially restricted.

Recent work sharpens this concept by identifying the intestine not only as an epithelial and microbial initiator organ but also as an immune initiator organ [85]. Ageing reshapes adaptive immunity such that lymphocytes progressively lose homeostatic and reparative functions while gaining activities that promote chronic inflammation, senescence and tissue dysfunction. This transition appears particularly consequential in the gut, where immune cells are required to maintain epithelial integrity, barrier selectivity and host–microbiota symbiosis [86].

Consistent with this view, senescence of gut germinal-centre B cells has been shown to arise during ageing in response to persistent commensal stimulation, leading to impaired IgA responses and altered control of the intestinal microbiota [87], with a comparable B-cell senescence shaping the oral microbiota [88]. Thus, immune senescence in the gut can act upstream of dysbiosis rather than simply resulting from it. In parallel, dysfunction of intestinal CD4 T-cell compartments has been shown to alter host–microbiota symbiosis, reduce regulatory and helper T-cell functions, increase inflammatory and cytotoxic T-cell states, and promote intestinal inflammation, barrier dysfunction, systemic inflammaging and senescence [86,89]. Importantly, restoration of CD4 T-cell function or microbiota depletion mitigates these phenotypes, indicating that adaptive immune dysfunction is sufficient to initiate pathological communication from the gut to the rest of the organism [89].

### (b) From immune dysfunction to vascular and barrier ageing

This mobile immune state has major consequences for the vasculature. Pro-inflammatory monocytes, macrophages and activated lymphocytes promote endothelial dysfunction by increasing oxidative stress, elevating adhesion molecules, impairing nitric oxide bioavailability and reinforcing SASP-like programmes in endothelial and perivascular cells [90]. Over time, this favours endothelial senescence, vascular stiffening, pro-thrombotic remodelling and microvascular rarefaction [91]. Specialized endothelial barriers are also vulnerable: at the blood–brain barrier (BBB), age-associated immune activation disrupts junctional integrity and promotes neuroinflammation; at renal, pulmonary and intestinal barriers, similar processes increase permeability and compromise tissue-specific homeostasis [92]. Thus, immune ageing helps translate upstream organ dysfunction into generalized barrier failure.

### (c) Endocrine, metabolic and neuroimmune circuits

Immune ageing is inseparable from endocrine and metabolic communication. Signals from adipose tissue, liver, muscle, pancreas and gut shape immune-cell development and activation, while aged immune cells reciprocally alter insulin sensitivity, tissue metabolism and ‘organokine’ output [93]. Human ageing-clock studies repeatedly identify immune and metabolic pathways as the signatures shared by organs that are ageing fastest. These data are correlative and do not by themselves establish transmission; what they show is that the same small set of outputs recurs whichever organ is affected, which is what the framework predicts if distinct organs converge on a common communicator trunk [94–96]. The hypothalamus appears to occupy a privileged position within this module [97]. Neuroendocrine centres integrate peripheral nutrient and inflammatory information and redistribute it through autonomic and hormonal outputs that influence adipose tissue, liver, vasculature and immune function placing central metabolic control within the communicator layer [98,99].

Neuroimmune communication provides a faster and more anatomically coordinated route of systemic ageing than blood-borne signals alone. Peripheral organs are densely innervated, and afferent neurons carry information about nutrient status, inflammation, injury and barrier dysfunction that is integrated in brainstem and hypothalamic circuits [100]. Efferent sympathetic, parasympathetic and neuroendocrine outputs then reshape immune-cell mobilization, vascular tone, metabolic allocation and tissue repair [101]. In ageing, these circuits are progressively shifted towards chronic sympathetic dominance and impaired anti-inflammatory restraint, favouring hypertension, marrow skewing and persistent low-grade inflammation [102].

The brain itself should therefore be assigned a dual role: it is a communicator, in that its neuroendocrine and autonomic outputs reshape peripheral physiology, and simultaneously an effector, in that it is among the tissues most sensitive to peripheral inflammatory input. This is not a contradiction but the expected consequence of axis-relative role assignment (box 1). Ageing of the neurovascular unit and BBB lowers the threshold for peripheral cytokines and immune cells to influence central function, while central inflammatory and neuroendocrine changes feed back to reshape peripheral physiology [103]. Once immune dysfunction and endothelial senescence compromise the BBB, peripheral inflammatory signals gain enhanced access to the central nervous system, amplifying microglial activation and hypothalamic dysregulation [97]. Similar logic may apply to other endothelial barriers, where neuroimmune dysfunction worsens local permeability and further increases exposure to circulating inflammatory cues [104]. The result is a feed-forward loop in which neural, immune and vascular communication progressively lose their buffering capacity.

## 5. Convergent effectors and breaks to organ communication

### (a) Shared effector processes across organs

In our hypothesis, initiator processes may differ in the nature, timing and severity of their primary lesions. Yet, this heterogeneity is communicated to the organismal tissues through sterile inflammation and converges on a restricted set of effectors that propagate dysfunction across target organs (figure 3). These effectors align with canonical hallmarks of ageing but can be hierarchically organized into cellular triggers and higher-order tissue responses.

**Figure 3.**
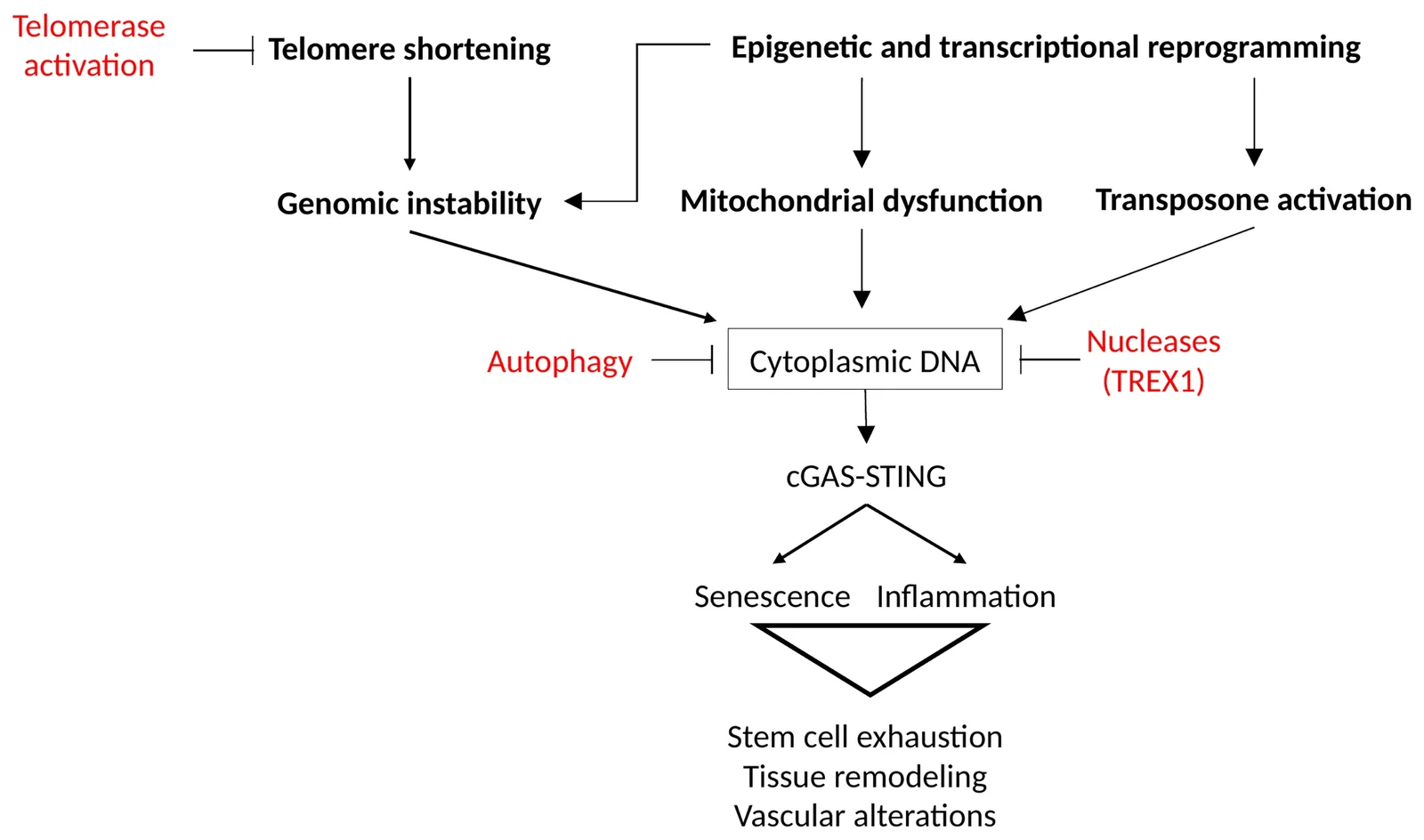
Integrated view of multiple initiator organs feeding into common effector processes; overlay key intervention points (telomerase, cGAS–STING, microbiota, autophagy, etc.).

At the cellular level, mitochondrial dysfunction represents an early and central hub. It is also the clearest illustration of role-relativity in this framework: mitochondrial dysfunction satisfies the initiator criteria of box 1 in muscle and adipose tissue, yet operates as an effector in organs whose bioenergetic decline follows the arrival of inflammatory and interferon signals. Ageing tissues exhibit progressive impairment of oxidative phosphorylation, accumulation of mitochondrial DNA (mtDNA) mutations, an increase in ROS production and loss of metabolic flexibility [105]. While adaptive responses, such as the mitochondrial integrated stress response and activation of selective macroautophagy, namely mitophagy, initially buffer these insults, their progressive failure with age leads to the release of mitochondrial DNA, RNA and other mitochondrial-derived danger signals. These further activate innate immune pathways, coupling bioenergetic decline to sterile inflammation [106,107].

In parallel, chronic metabolic and inflammatory stresses drive epigenetic and transcriptional reprogramming, which stabilize ageing-associated phenotypes and promote genomic instability [108]. Age-associated changes in DNA methylation, histone modifications, and chromatin accessibility and heterochromatin integrity reinforce pro-inflammatory and senescent gene expression programmes. These alterations increase transcriptional noise, activate transposable elements and impair DNA damage responses, thereby creating a durable molecular memory of ageing within tissues [2,108,109].

These molecular alterations converge at the tissue level to drive stem and progenitor cell exhaustion, microenvironmental remodelling and vascular decline [110]. Across multiple organs, chronic mitochondrial stress and epigenetic drift reduce stem-cell self-renewal capacity, bias lineage commitment, disrupt niche architecture and impair regeneration, often through non-cell-autonomous mechanisms mediated by systemic inflammation, rather than intrinsic damages [111–114]. In parallel, endothelial senescence, fibrosis and extracellular matrix remodelling compromise tissue perfusion and barrier function [115–117].

Importantly, these tissue-level effectors can be induced in secondary organs that have not yet accumulated substantial local damage [21,67,118]. This dissociation between intrinsic lesion burden and functional decline supports therapeutic strategies aimed at interrupting persistent transmission or interpretation of ageing signals, rather than attempting to correct cell-autonomous damage across all tissues.

### (b) Nucleases and autophagy as brakes on sterile inflammation

Within this framework, nucleic acid clearance pathways and autophagy serve as key intrinsic brakes on sterile inflammation by limiting the accumulation of immunostimulatory debris [119,120]. Cytoplasmic DNases and RNases, such as TREX1, DNaseII, ANKLE1 and RNase H2, degrade aberrant DNA and RNA species that would otherwise engage cGAS–STING and related pathways [121,122]. Genetic or functional impairment of these nucleases, as well as their downregulation in senescent cells, promotes cytoplasmic nucleic acid accumulation, chronic inflammatory signalling and SASP [58,60,64,123]. Upregulation of TREX1 has been recently shown to reduce SASP and limit senescence *in vitro* [53,58,124]. Because nucleic-acid-induced sterile inflammation appears central to ageing, strengthening these clearance systems is a promising way to restrain pathological communication.

Autophagy and mitophagy are a second major brake on age-associated inflammation because they clear damaged mitochondria, cytosolic nucleic acids and other DAMPs, thereby restraining inflammasome activation and SASP [125–127]. Nucleic acid sensing and autophagy are functionally intertwined, since cGAS- and STING-dependent programmes can promote autophagic clearance, while finely tuning the immune response by controlling STING degradation [128–130]. Moreover, active TBK1, a downstream cGAS–STING effector, is responsible for mitophagy activation [131,132].

With ageing, autophagic flux and lysosomal competence decline across multiple tissues. This enables the persistence of damaged organelles and immunostimulatory nucleic acids and creates a permissive environment for chronic, low-grade innate immune activation [133,134]. Therefore, the progressive erosion of these clearance systems represents a key enabling mechanism of inflammaging, mechanistically linking molecular damage accumulation to sustained inter-organ inflammatory communication.

### (c) Evidence for convergence from organ-specific interventions

Compelling evidence for convergence on shared effector pathways comes from interventions that target single initiating organs yet elicit coordinated improvements across distant tissues. The gut-restricted telomerase experiment described in §3b is the clearest vertebrate case [67]: loss of cGAS–STING signalling produces a comparable rescue of organ damage and lifespan [39], indicating that much of the effector-layer pathology is driven by communicated signals rather than local telomere status alone.

Comparable single-organ interventions have been described in worms, flies and mice. The *Caenorhabditis elegans* tissue-specific manipulation of insulin/IGF signalling provided the foundational idea: global, intestinal- or neuronal-specific loss of the insulin/IGF receptor DAF-2 extends lifespan [135]. Contrarily, restoring daf-2 selectively in neurons on DAF-2 mutant worms largely suppresses this longevity extension, demonstrating that insulin signalling in a single tissue can override systemic communication and set the organismal ageing rate [135–137]. Parallel phenomena are observed in *Drosophila melanogaster*. Increasing mitochondrial biogenesis in intestinal stem cells or activating AMPK–Atg1-dependent autophagy specifically in gut epithelium slows systemic ageing [138,139]. Conversely, silencing autophagy-related genes in adipocytes or gut cells reduced lifespan [140]. Likewise, activation of FOXO restricted to skeletal muscle or the fat body, the fly analogue of mammalian liver/white adipose tissue, preserves proteostasis, enhances autophagy, confers organism-wide stress resistance and extends lifespan [141–143]. Similarly, the overexpression of the key activator of the endoplasmic reticulum unfolded protein response (UPR^ER^), Xbp1, in the fat body or the gut increases mitochondrial activity and improves tissue homeostasis with similar longevity benefits [144]. Finally, in mice, genetically, fasting- or exercise-induced autophagy in liver or skeletal muscle improves whole-body glucose and lipid homeostasis, again showing that repairing a single metabolic organ can reprogramme systemic energy balance and indirectly protect remote tissues [145–147]. Moreover, the restoration of synaptic functionality in the hypothalamus affects neural–fat tissue communication and ameliorates white adipose tissue function and metabolic activity, extending lifespan [148].

Together, these interventions support a general principle: shared effector mechanisms in secondary organs, such as stem-cell failure, mitochondrial dysfunction, epigenetic drift and tissue remodelling, can be significantly shaped and, in some cases, reversed by manipulating a limited number of organs or communication nodes even when primary initiators are only partially corrected.

## 6. Open questions and future directions

### (a) One initiator or multiple?

Human organ-specific epigenetic and proteomic clocks extend this picture: arteries, brain, kidney, liver, immune system or gut may be biologically older or younger in different individuals, yet acceleration in these organs predicts disease and multimorbidity and is repeatedly associated with immune and metabolic pathway signatures [21,22,94]. Rather than a collection of unrelated causes, these findings are better interpreted as multiple entry points into the same communication framework [21,94,149].

The practical problem then becomes which organ(s) cross a critical damage threshold first. How stable is that dominance over time, and can interventions that stabilize one organ (e.g. gut, vasculature or brain) reorder the hierarchy so that other tissues remain functionally younger despite accumulating initiator lesions of their own? Addressing this will require longitudinal, organ measurements of both damage and systemic communication, not just glimpses of moments in time.

### (b) A common communicator ‘trunk’?

Across species and organs, the same types of outputs recur when a single organ is manipulated. Despite different initiators and anatomical sites, these interventions converge on a limited set of systemic mediators: inflammatory cytokines, interferons, senescence-associated factors, metabolic hormones and neuroendocrine outputs [7,149].

These observations can be interpreted by a common ‘fan-in/fan-out’ communicator trunk (figure 1). Initiator organs (gut, vasculature, kidney, liver, brain and adipose) feed into a relatively small set of communicator modules, such as sterile inflammatory (including SASP), interferon and nucleic acid sensing pathways, endocrine and metabolic hormones, and neuroimmune outputs. These modules are then broadcast back out to several tissues, where local context determines whether the same signal elicits repair, compensation or degeneration. The initiator–communicator–effector model thus provides a unifying language: ageing phenotypes in different organs are not independent, but different projections of a shared communicator trunk onto tissue-specific effector landscape.

It should be emphasized that this is a directed architecture, and that directedness is not acyclicity. Effector states feed back onto the initiator layer: fibrosis and matrix stiffening reduce perfusion and thereby promote the mitochondrial failure and mitochondrial DNA release that engage cGAS–STING, while myeloid-biased haematopoiesis returns inflammatory input to the mucosa whose barrier failure initiated it. The framework therefore describes a directed cyclic graph in which the biologically informative quantities are edge weight and latency rather than the mere presence of connections, and in which linear, tree-like and reticulate descriptions each capture part of the structure [150]. Of the three, the tree-like description fits our data best, and figure 1 is drawn accordingly: many organ-intrinsic initiators converge on a shared communicator trunk, from which a smaller set of effector states radiates. The architecture is neither one linear chain nor an undifferentiated network but many linear axes superposed on a common trunk. That is what gives the framework multiple entry points while keeping a direction of flow that can still be found. Latency carries information for the same reason: the same output can protect or destroy according to how long it runs, since a transient burst recruits repair and clearance, whereas the same signal sustained daily pushes a tissue into chronic interferon exposure and reinforced senescence. The ordering we propose is a claim about which edges dominate on a given axis, in a given individual, at a given time, and it is empirically decidable: gut-restricted telomerase re-expression improves gonadal and haematopoietic outcomes, while telomeres in those organs remain short [67], an asymmetry that a purely reticulate description accommodates but does not predict. Formalizing the framework as a weighted graph is also what would allow hubs to be identified and ranked by network-centrality methods, which is the analytical basis of the translational priorities set out below. Testing the framework as a whole is a different matter. Because roles are axis-relative, any one contradictory result can be reassigned to another axis, so refutation has to rest on predictions made jointly across axes. The architecture makes one such prediction: correcting a dominant initiator organ should protect several untreated recipients, whereas correcting a downstream recipient should not. Comparing gonad- or marrow-restricted telomerase re-expression with the gut-restricted rescue of §3b would test it, and equivalent benefit from either would refute the trunk, with no axis left to retreat to. Refuting it quantitatively would mean fitting the weighted graph against symmetric, reversed and fully distributed alternatives on longitudinal, organ-resolved data.

### (c) Vectors of transmission: how communicator signals travel

The preceding sections identify what is broadcast; they do not specify how it moves. Five physical modes carry the signals discussed here, and they differ in range, latency and specificity. First, free soluble factors, namely cytokines, interferons, hepatokines, myokines, adipokines, microbial metabolites and hormones, travel in the portal and systemic circulation, the mode most directly demonstrated by heterochronic parabiosis and plasma-transfer experiments. Second, extracellular vesicles package proteins, lipids, metabolites and nucleic acids within a lipid bilayer, conferring protection from extracellular nucleases and proteases, extended half-life and, through their surface composition, a degree of tissue tropism and a traceable tissue of origin; their cargo overlaps extensively with the SASP and with the nucleic acid species central to sterile inflammation [151–155]. Third, circulating cells act as mobile carriers, transporting transcriptional and epigenetic states acquired in one tissue to another (§4a). Fourth, neural conduction provides a fast, non-humoral channel through autonomic and sensory afferents (§4c). Fifth, physical routes, such as altered perfusion, matrix stiffening and lymphatic drainage, modify the delivery of all of the above.

This distinction matters most for the nucleic acid arm of the model. Cytosolic DNA is by definition intracellular, and its sensing would remain confined to the cell of origin were the second messenger not transmissible. 2′3′-cGAMP passes between cells through gap junctions [156], is imported by the folate transporter SLC19A1 [157,158] and by LRRC8-containing volume-regulated anion channels [159] and is carried within extracellular vesicles. Cell-free mitochondrial and nuclear DNA, and micronuclear fragments released from dying cells, likewise circulate and increase with age. These routes are what convert a cell-intrinsic sensing pathway into an organ-to-organ one, and they are correspondingly the points at which the interferon arm of the framework might be interrupted pharmacologically without abolishing intracellular cGAS–STING function.

### (d) Evolutionary considerations

Organ communication systems did not evolve to accelerate ageing. Rather, they have evolved to coordinate development, reproduction, metabolism and defence from pathogens. A definition is required before this argument can be made. Transmission between organs is asymmetric with respect to evolutionary design. Some routes are communication in the strict sense: matched ligand–receptor systems with dedicated secretory and transduction machinery, selected to convey information between tissues, as in endocrine axes and the inflammatory reflex. Others are spillover, in which nothing on the sender side evolved to transmit, such as barrier leakage, release of mitochondrial DNA from failing organelles, altered perfusion. The receiver, however, is almost invariably evolved to interpret: cGAS, TLR4 and NLRP3 are dedicated sensors selected for precisely these ligands. Ageing exploits this asymmetry, in which unselected emissions are read by selected receivers. We retain the term communication for both classes because the systemic consequence is the same, while noting that only the first carries an evolved signalling function. Nor should any of this be read as a claim that every interdependence between organs is an adaptation: some coupling is a by-product of shared vasculature, common developmental origin or simple spatial adjacency, carries no selected function at any age and can transmit dysfunction just as effectively as an evolved axis [160]. Many pathways highlighted here play clearly adaptive roles in early life. Their late-life contribution to pathology likely reflects a combination of cumulative damage, loss of regulatory brakes and the relaxation of selective pressure after reproduction. Human proteomic maps of ageing consistently implicate innate immune signalling, extracellular matrix remodelling and synaptic-glial interactions [21]. This indicates that pathways governing host defence and tissue repair, when chronically engaged, may contribute to the propagation of age-related dysfunction rather than merely reflecting it.

This perspective suggests that ageing is, in part, a problem of signal persistence and timing [161]. Mechanisms that are beneficial when transient become harmful when chronically engaged; we use maladaptive throughout in this restricted, antagonistic-pleiotropy sense, to denote a mechanism whose fitness benefit is confined to acute or early-life contexts and whose late-life persistence is deleterious [160], rather than to assert that the mechanism is currently under negative selection. The p53 response is the familiar instance: measured activation after DNA damage enables arrest and repair, whereas sustained activation drives tissue attrition, and organs that are efficient communicators in the young become sources of harmful signalling when damaged. Evolutionary arguments therefore support targeting the dynamics of these systems, such as shortening the duration and reducing the activation, rather than abolishing them completely.

### (e) Translational implications and priorities

If organ-to-organ communication is central to systemic ageing, interventions should first identify which organs act as dominant initiators in a given context. Severe single-lesion models are informative because they confine damage in space and time, which is what the criteria of box 1 demand. Physiological ageing is less obliging. Whether it too has temporally dominant initiator organs, or instead several weak and shifting entry points, is unsettled, and organ-resolved clocks are only now making the question answerable. In humans, this hierarchy can be inferred by combining organ-resolved epigenetic clocks with organ-specific proteomic clocks, plus imaging and functional tests [21]. Together, these read-outs report not only which organs are biologically older but also which are most actively reshaping systemic ageing.

Once initiators are identified, therapies can be refocused on stabilizing these organs and modulating their main communicator signals instead of attempting whole-organism rejuvenation. This is exemplified by strategies that, applied to humans, target barrier, vascular, metabolic or neural hubs and selectively dampen their dominant inflammatory or endocrine communicator signals, rather than applying broad immunosuppression or systemic growth-factor inhibition [162,163]. In parallel, reinforcing endogenous brakes, such as autophagy, nucleic acid clearance and selective elimination or reprogramming of high-output senescent cells, offers a way to reduce the amplitude and duration of pathological communication without abolishing acute host-defence functions. Concretely, restoring mitophagy, stabilizing micronuclear envelopes or re-establishing heterochromatin lowers the nucleic acid burden that feeds the sensing pathway, attenuating a communicator by reducing its input rather than by blocking it and sparing the surveillance functions that outright blockade removes [164].

Progress will also depend on biomarkers that report communication activity, not just local damage. Organ-specific epigenetic and proteomic clocks, together with tissue-linked ageing signatures and plasma-tissue paired measurements, should provide non-invasive read-outs of which organs are ageing fastest and how strongly they broadcast signals into circulation. Combined with longitudinal and functional data, such markers will allow stratification by dominant communication states and real-time assessment of whether an intervention truly protects secondary organs from an initiating lesion.

## Data Availability

This article has no additional data.

## Theme

One contribution of 21 to a theme issue ‘Developmental circuits in ageing’.
